# Detection of arboviruses in mosquitoes: Evidence of circulation of chikungunya virus in Iran

**DOI:** 10.1371/journal.pntd.0008135

**Published:** 2020-06-30

**Authors:** Hasan Bakhshi, Laurence Mousson, Sara Moutailler, Marie Vazeille, Géraldine Piorkowski, Sedigheh Zakeri, Abbasali Raz, Xavier de Lamballerie, Navid Dinparast-Djadid, Anna-Bella Failloux

**Affiliations:** 1 Malaria and Vector Research Group, Biotechnology Research Center, Pasteur Institute of Iran, Tehran, Iran; 2 Institut Pasteur, Arboviruses and Insect Vectors, Paris, France; 3 UMR BIPAR, Animal Health Laboratory, ANSES, INRA, Ecole Nationale Vétérinaire d’Alfort, Université Paris-Est, Maisons-Alfort, France; 4 Unité des Virus Emergents (UVE), Aix Marseille Université, IRD 190, INSERM 1207, IHU Méditerranée Infection, Marseille, France; DoD - AFHSB, UNITED STATES

## Abstract

Mosquitoes are vectors of viruses affecting animal and human health. In Iran, the prevalence of mosquito-borne viruses remains poorly investigated. Once infected, mosquito females remain infected for all their life making virus detections possible at early steps before infections are reported in vertebrate hosts. In this study, we used a recently developed high-throughput chip based on the BioMark Dynamic arrays system capable of detecting 37 arboviruses in a single experiment. A total of 1,212 mosquitoes collected in Mazandaran, North-Khorasan, and Fars provinces of Iran were analyzed. Eighteen species were identified, belonging to five genera; the most prevalent species were *Anopheles maculipennis* s.l. (42.41%), *Culex pipiens* (19.39%), *An*. *superpictus* (11.72%), and *Cx*. *tritaeniorhynchus* (10.64%). We detected chikungunya virus (CHIKV) of the Asian genotype in six mosquito pools collected in North Khorasan and Mazandaran provinces. To our knowledge, this is the first report of mosquitoes infected with CHIKV in Iran. Our high-throughput screening method can be proposed as a novel epidemiological surveillance tool to identify circulating arboviruses and to support preparedness to an epidemic in animals and humans.

## Author summary

Mosquito-borne viruses pose a serious threat to public health worldwide. For an efficient preparedness to future emergences, an early detection of arboviruses in vector populations would be wise before animal/human cases arise. Here, we used a recently developed high-throughput chip based on the BioMark Dynamic arrays system, capable of screening various arboviruses in a single experiment. By examining 1,212 mosquitoes collected in Iran, we provide evidence that chikungunya virus is circulating in Iran.

## Introduction

Up to date, 64 mosquito species are recorded in Iran [1, 2] and several cryptic species have been identified using molecular tools [3, 4]. Mosquitoes of the main genera, *Culex* and *Aedes* are incriminated as the most important vectors of arboviruses [5, 6]. The increasing volume of people displacements including movements of refugees, and the traffic of goods and animals between countries of the region, offer multiple opportunities for arbovirus introduction in Iran such as dengue virus (DENV), and West Nile virus (WNV) [7, 8].

WNV is the most prevalent *Culex-*transmitted virus frequently reported in Iran [9, 10]. Humans [11] and horses [12] are common vertebrate hosts. Migratory birds play a critical role in introducing WNV; 15% were found serologically WNV-positive and among them, 54% concerned *Fulica atra* birds suggesting the potential role of common coots in WNV ecology in Iran [13]. Chinikar et al., in 2012 and 2013, found that three patients developing encephalitis were positive for WNV by RT-qPCR assay [14] and detected serologically positive sera in 1.3% of humans and 2.8% of equines [15]. WNV was also detected in mosquitoes: *Aedes caspius* in the Northwest [16], and *Culex pipiens* in the North [17] and the South [8]. Besides, the most critical mosquito-borne virus in the world is DENV mainly transmitted between humans by *Aedes* mosquitoes [18]. Most human cases in Iran were reported in the southeast of the country near the border with Pakistan [7, 19–21], though local transmission has not been confirmed yet [22]. In addition, circulation of chikungunya virus (CHIKV) was suspected in Pakistan [23, 24], possibly due to the global expansion of CHIKV since 2005 [25]. Then, to the best of our knowledge, there is no report of other mosquito-borne viruses like Zika virus (ZIKV), yellow fever virus (YFV) and CHIKV in Iran. Early detection of arboviruses in mosquitoes is a pre-requisite for designing and implementing adapted control measures. To improve the surveillance of arboviruses circulating in humans and animals, new molecular tools are required to screen a wider panel of arboviruses. A novel high-throughput epidemiological surveillance method developed by [26] has been designed using a microfluidic system (BioMark dynamic array system, Fluidigm) capable of performing parallel real-time PCRs using 96.96 chips resulting in 9,216 individual reactions; the original design concerned 149 primers/probe sets able to detect 59 viruses (different genotypes/serotypes) which was validated on experimentally infected mosquitoes and tested using field-collected mosquitoes. Our study aims at identifying arboviruses belonging to the *Flaviviridae*, *Togaviridae* and *Bunyaviridae* families in mosquitoes collected in Iran using this newly developed tool.

## Methods

### Study area, field collections and identification of samples

**Fig 1:**
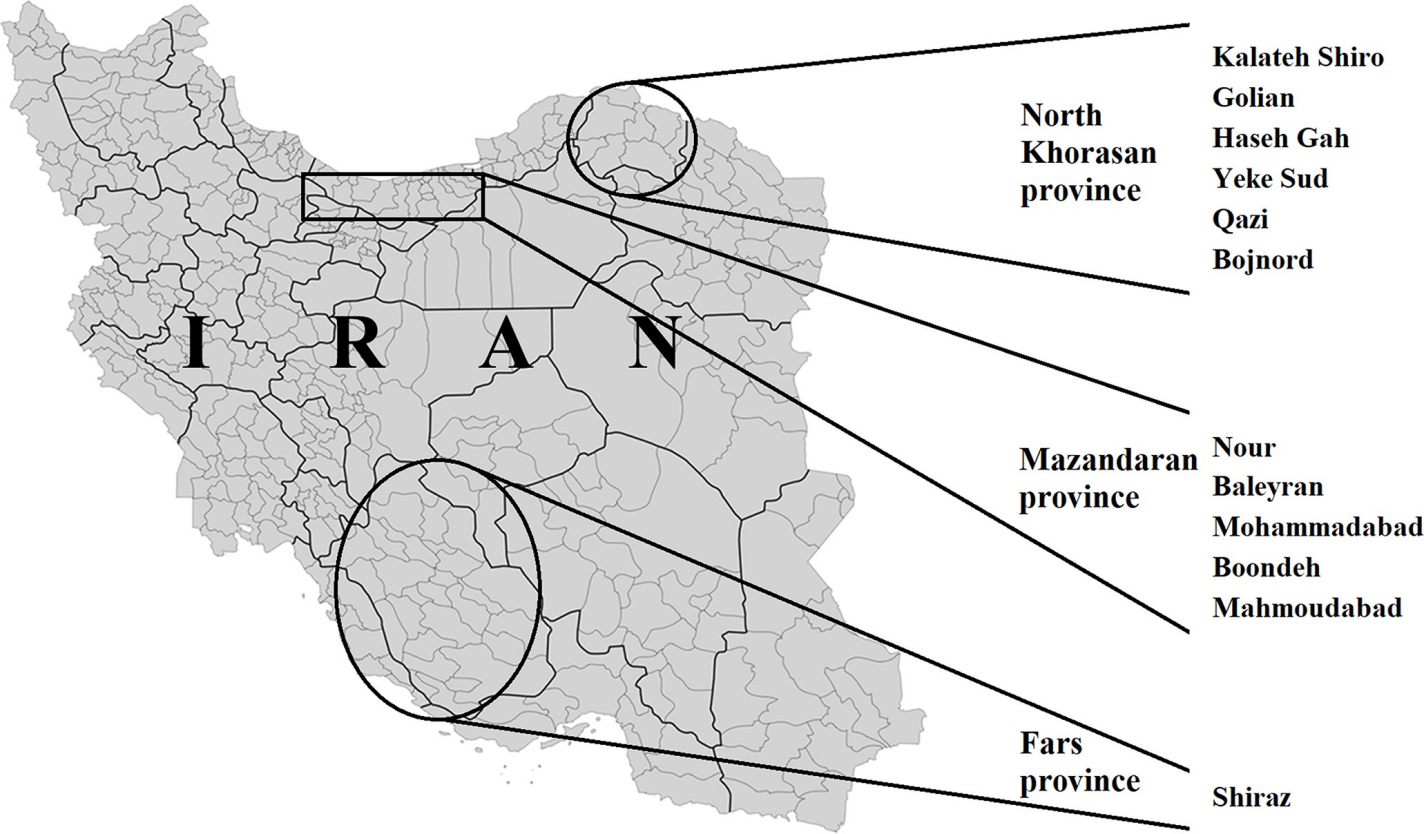
Map of mosquito collection sites from North Khorasan, Mazandaran, and Fars provinces in Iran. The map was built using the open source map site “https://commons.wikimedia.org/wiki/File:Iran_Counties.svg”.

Based on the important agricultural, husbandry, business, and industrial activities, three provinces were selected for mosquito collections: North Khorasan, Mazandaran, and Fars. North Khorasan province with a moderate highland climate in general (covering an area of 28,434 km^2^), is surrounded by Republic of Turkmenistan on the north, Razavi Khorasan province on the east and south, Golestan province on the west and Semnan province on the south western part; the capital of the province is Bojnord. Mazandaran province with a moderate subtropical climate (covering an area of 23,842 km^2^), is located along the southern coast of the Caspian Sea and is bordered by Russia across the sea, Golestan, Semnan, Tehran, Alborz, Qazvin, and Guilan provinces. The diverse nature of the province mostly features rice fields, prairies, forests and rainforest; Sari is the capital of the province. Fars Province with an area of 122,400 km^2^ is located in southwest Iran, and Shiraz with a population of 1,869,000 is its administrative center. The climate of Shiraz has distinct seasons, and is overall classed as a hot semi-arid climate. Adults were collected during July-September 2018 corresponding to the season of high risk of transmission with high mosquito densities and presence of vertebrate hosts such as migratory birds. Night-biting mosquitoes were caught using CDC light traps (two repetitions; traps were not baited with CO_2_, and were placed in front of houses and barns from 7:00 PM to 7:00 AM) and day-biting mosquitoes were sampled using aspirators to capture adults when landing on human and animal baits. Human landing catches were conducted in sites where there were no reports of *Aedes*-vectored disease cases during the period of mosquito collections. The duration of collection was 2 hrs in each collection site and mosquito morphological identification was based on the key of Azari-Hamidian and Harbach [27]. Subsequently, mosquitoes were dissected to separate abdomen from the remaining parts of body (RPB). Abdomens were grouped by species, location and pools of 10, and RPB were stored individually. If a virus was detected in a pool, screening of corresponding individual RBPs was performed to define mosquito infection rates. Regarding North Khorasan province, sampling was conducted in six districts within four counties: Kalateh Shiro, Golian, Haseh Gah, Yeke Sud, Qazi, and Bojnord. For Mazandaran province, samples were collected in five districts within three counties: Baleyran, Nour, Mohammadabad, Boondeh, and Mahmoudabad. Regarding Fars province, sampling was provided only from Shiraz, the capital city (Fig 1). A total of 122 pools and 1,212 mosquitoes were screened.

### Detection of arboviruses

Total RNA was extracted from pools of abdomens with the Nucleospin RNA extraction kit (Macherey-Nagel, Hoerdt, France) and retro-transcribed using the qScript cDNA Supermix kit according to the manufacturer’s instructions (Quanta Biosciences, Beverly, USA). Obtained cDNAs were pre-amplified with the Perfecta Preamp Supermix (Quanta Biosciences, MA, USA) kit according to the manufacturer’s instructions. Briefly, all primers were pooled to 200 nM final each. The reaction was performed in a final volume of 5 μL containing 1 μL Perfecta Preamp 5X, 1.25 μL pooled primers, 1.5 μL distilled water and 1.25 μL cDNA, with one cycle at 95°C for 2 min, 14 cycles at 95°C for 10 sec and 3 min at 60°C. At the end of the cycling program, the reactions were 1:5 diluted. cDNAs were tested in the chip based on the BioMark Dynamic arrays system (Fluidigm Corporation); it allows high-throughput microfluidic real-time PCR amplifications in one experiment using 96 PCR mixes and 96 samples [28]. Amplifications were performed using 6-carboxyfluorescein (FAM)- and black hole quencher (BHQ1)-labeled TaqMan probes with TaqMan Gene expression master mix (Applied Biosystems, France). Thermal cycling conditions were: 2 min at 50°C, 10 min at 95°C, followed by 40 cycles of 2-step amplification of 15 sec at 95°C, and 1 min at 60°C. Data was acquired on the BioMark Real-Time PCR System and analyzed using the Fluidigm Real-time PCR Analysis software to obtain crossing point values. One negative water control was included per chip. To determine if inhibitors present in the sample can inhibit the real-time PCR, a strain of *Escherichia coli* was added to each sample as an internal inhibition control. Among the 149 primers/probe sets developed in Moutailler et al. (2019) [26], 95 sets targeting 95 different genotypes/serotypes of 37 viral species were chosen for our screening (design of primers/probe sets are available in S1 Table from [26]). Primers and probes were tested and only few cross reactions were observed between serotypes and/or genotypes of targeted viruses (i.e. cross reactions between dengue serotypes, between Usutu virus and WNV). Once the virus was detected in pools of mosquito abdomens, a screening of corresponding individual RBPs was performed to define mosquito infection rates. The virus genotype was defined by RT-PCR and virus isolation was attempted on insect C6/36 cells.

## Results

### Diversity of mosquito species in the study area

**Fig 2:**
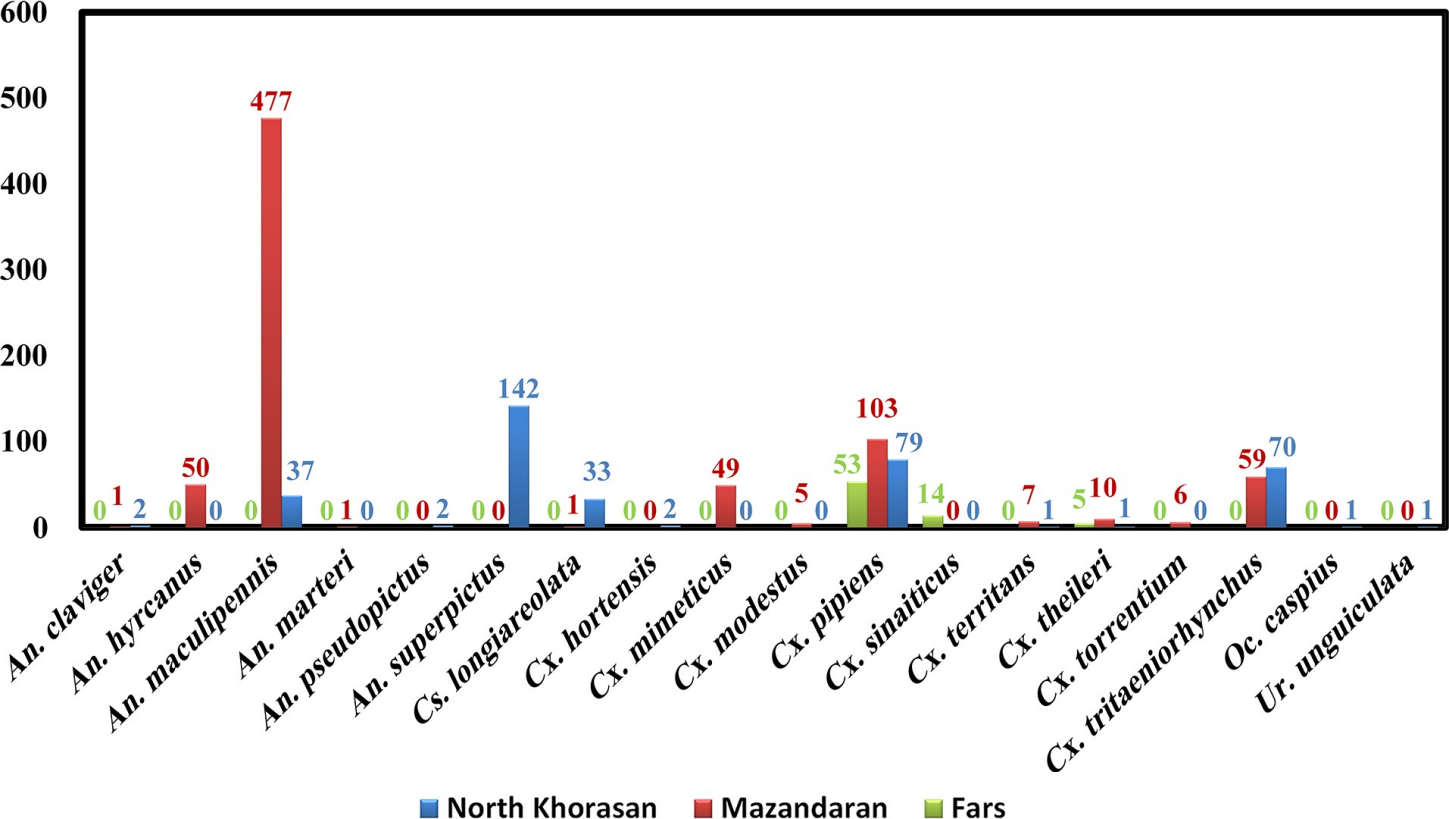
Diversity of mosquito species collected. Out of 1,212 caught mosquitoes, 18 species belonging to five genera were identified.

A total of 1,212 samples were processed: 371, 769, and 72 mosquito female samples from North Khorasan, Mazandaran, and Fars provinces respectively (S1 Table). Morphological identification revealed the presence of 18 species belonging to five genera in the study area. The most prevalent species belonged to *Anopheles maculipennis* s.l. (42.41%), *Cx*. *pipiens* (19.39%), *An*. *superpictus* (11.72%), and *Cx*. *tritaeniorhynchus* (10.64%). *An*. *hyrcanus* (4.12%), *Cx*. *mimeticus* (4.04%), *Culiseta longiareolata* (2.8%), *Cx*. *theileri* (1.32%), *Cx*. *sinaiticus* (1.16%), *Cx*. *territans* (0.67%), *Cx*. *torrentium* (0.49%), *Cx*. *modestus* (0.41%), *An*. *claviger* (0.25%), *An*. *pseudopictus* (0.17%), *Cx*. *hortensis* (0.17%), *An*. *marteri* (0.08%), *Ochlerotatus caspius* (0.08%), and *Uranotaenia unguiculata* (0.08%) were also found (Fig 2).

### High-throughput screening of arboviruses

**Fig 3:**
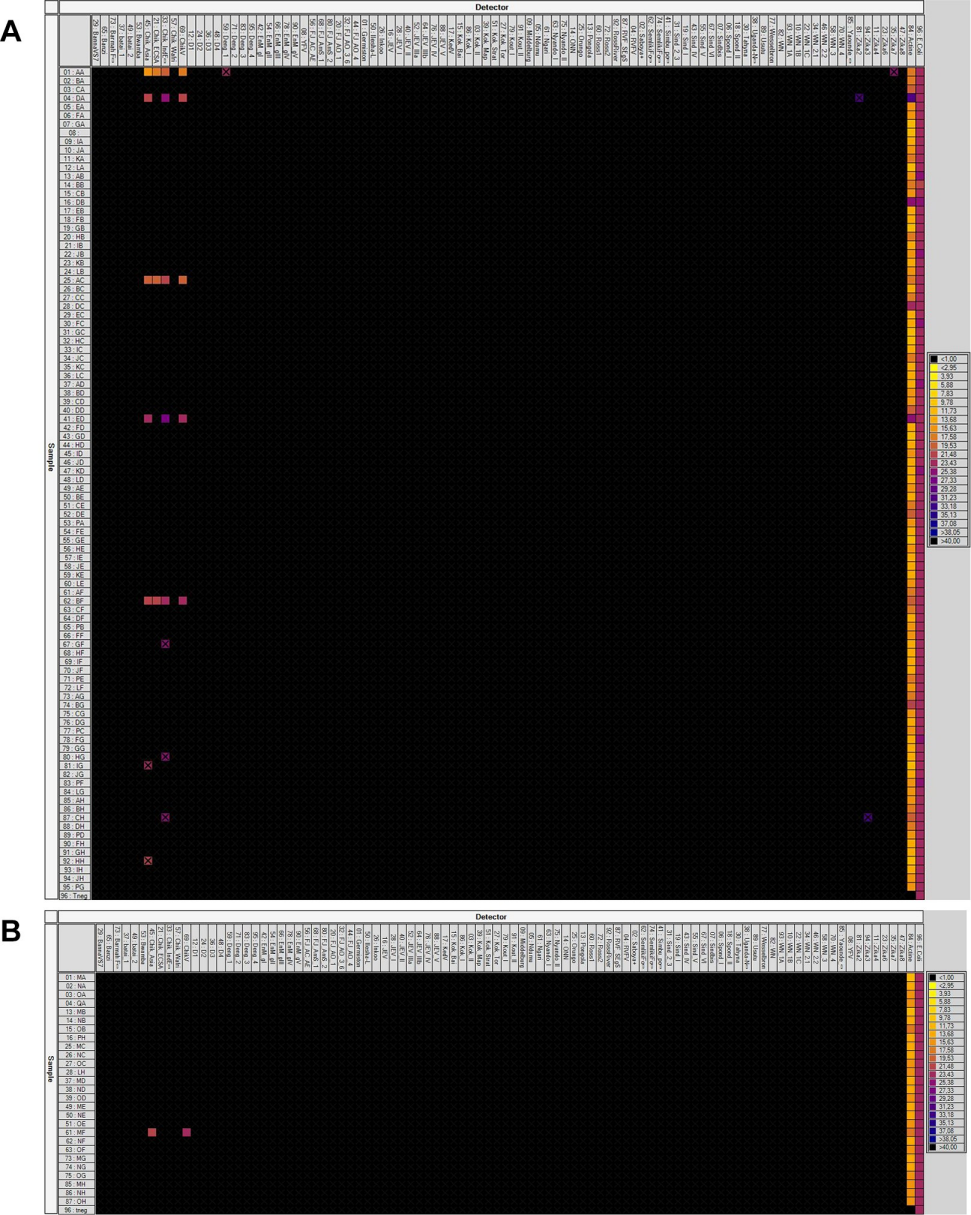
Screening of mosquitoes collected in Iran using the BioMark dynamic array system (96.96 chip). A. First batch of 95 mosquito pools. B. Second batch of 27 mosquito pools. Each square corresponds to a single real-time PCR reaction, where rows indicate batches of mosquitoes tested and columns represent the targets of each primer/probe set. Cross indicate cross-reaction of assays. Ct values for each reaction are indicated in color; the corresponding color scale is presented in the legend on the right. The darkest shades of blue and black squares are considered as negative reactions with Ct > 30. The 37 viruses targeted (95 primers/probe sets for different genotypes/serotypes) were: Banna; Banzi; Barmah Forest; Batai; Bwamba; Chikungunya (Chik_ECSA, Chik_IndECSA, Chik_Asia, Chik_Wafri, ChikV); Dengue (D1, D2 D3, D4, Deng_1, Deng_2, Den_3, Deng_4); Murray Encephalitis (EnM_gI, Enm_gII, EnM_gIII, EnM_gIV, EnM_gV); Yellow fever (YFV, FJ_AO_1, FJ_AO_3_6, FJ_AO_4, FJ_AC_AE, FJ_AmS_1, FJ_AmS_2); Germiston; Ilesha; Inkoo; Japanese Encephalitis (JEV, JEV_I, JEV_II, JEV_IIIa, JEV_IIIb, JEV_IV, JEV_V); Kedougou (KedV); Kokobera group (Kok_ I, Kok_II, Kok_Bai, Kok_Tor, Kok_Map, Kok_Strat); Koutango (Kout_I, Kout_II); Middelburg; Ndumu; Ngari; Nyando; O’nyong-nyong (ONN); Orungo; Pongola; RossRiver (RossRiver, Ross1, Ross2); Rift Valley Fever (RVFV, RVF_SegS); Saboya; Semliki forest; Simbu; Sindbis (Sindbis, Sind_I, Sind_2_3, Sind_IV, Sind_V, Sind_VI); Spondweni (Spond_I, Spond_II); Tahyna; Uganda; Usutu; Wesselsbron; West Nile (WN, WN_1A, WN_1B, WN_1C, WN_2.1, WN_2.2, WN_3, WN_4); Yaounde; Zika (Zika2, Zika3, Zika4, Zika6, Zika7, Zika8). Sequences of primers/probe sets are available in S1 Table of [26] or under request.

Viral detections using the BioMark microfluidic system, able to screen 37 viral species including 95 genotypes/serotypes (detail of viruses targeted presented in the legend of Fig 3), showed the presence of CHIKV in six pools collected from North Khorasan (5) and Mazadaran (1) provinces (Fig 3). When analyzing individual mosquitoes in CHIKV-positive pools, we found six CHIKV-infected individual head-thorax (Table 1). CHIKV was detected in single mosquitoes: *Cs*. *longiareolata* from Kalateh Shiro and Yeke Sud, *Cx*. *tritaeniorhynchus* from Kalateh Shiro and Golian, and *An*. *maculipennis* s.l. from Golian and Baleyran. *Cs*. *longiareolata* mosquitoes should be considered carefully as the pools contained engorged mosquitoes (i.e. having ingested a blood meal potentially infected). *Cx*. *tritaeniorhynchus* and *An*. *maculipennis* s.l. were detected positive with primers designed for the CHIKV Asian genotype. Attempts to isolate the virus failed and whole genome sequencing was not performed.

**Table 1:** Details of provinces, counties, and districts in Iran where mosquitoes were collected during July to September 2018.

Province	County	Place of collection	Coordinate	Altitude (m)	Pools CHIKV-infected	CHIKV-infected species
North Khorasan	Maneh and Samalqan	Haseh Gah	37°39′N 57°02′E	617	0	*-*
	Raz and Jargalan	Yeke Sud	38°10′N 56°39′E	938	1	*Cs*. longiareolata*
	Shirvan	Golian	37°13'N 57°53'E	1439	2	*Cx*. *tritaeniorhynchus* *An*. *maculipennis s*.*l*.
	Bojnord	Qazi	37°28′N 57°33′E	1130		
	Raz and Jargalan	Kalateh Shiro	37°57'N 56°59'E	1094	2	*Cs*. longiareolata* Cx. *triateniorhynchus*
	Bojnord	Bojnord	37°24′N 57°17′E	1187		
Mazandaran	Amol County	Baleyran	36°21′N 52°25′E	170	1	*An*. *maculipennis s*.*l*.
	Nour County	Nour	36°33′N 52°02′E	0	-	-
	Amol County	Mohammadabad	36°12′N 52°24′E	973	-	-
	Mahmoudabad	Boondeh	36°34′N 52°14′E	-12	-	-
	Mahmoudabad	Mahmoudabad	36°37′N 52°16′E	-15	-	-
Fars	Shiraz	Shiraz	29°35′N 52°32′E	1594	-	-

* Mosquitoes from a pool containing engorged females.

## Discussion

In our study, CHIKV was detected in six individual mosquitoes (6/1,212): five collected in North Khorasan province and one in Mazandaran province. Surprisingly, no other arboviruses were detected. This report of CHIKV circulation in mosquitoes suggests that this virus is present in Iran.

We used a newly developed high-throughput virus-detection assay based on microfluidic PCRs able to detect 64 mosquito-borne viruses in mosquitoes [26]. Among 149 primers/probe sets, only four primer sets showed cross-reactivity with viruses from the same genus or serotype. This tool has been validated on field-collected mosquitoes in a large epidemiological survey in six countries/territories during the last Zika pandemic; with 17,958 mosquitoes collected, three human infecting arboviruses, ZIKV, YFV, and CHIKV, and one rare arbovirus, Trivittatus virus, were detected. In our study, we used 95 primers/probe sets targeting the different genotypes/serotypes of 37 viruses to screen 1,212 collected mosquitoes. We were able to detect CHIKV in six pools and confirmed the result by classical RT-PCR in six individual head/thorax. We failed to isolate the virus and to perform whole genome sequencing. The infection rate of 0.5% in mosquitoes we have obtained is lower than what could be estimated during an epidemic [29]. However this screening method can be used as a passive surveillance system alerting of any increase in infection rates in mosquitoes, sign of an imminent epidemic. In our study, we used CDC light traps and aspirators to collect night-biting and day-biting mosquitoes respectively, and were able to capture 1,212 adult females. We suggest using complementary collection tools (such as BG-sentinel traps and gravid female traps) covering more collection sites to get more chance to detect arboviruses in collected samples including *Aedes* mosquitoes. A growing body of evidence supports that arboviruses are circulating in Iran. WNV was detected in mosquito vectors as well as vertebrate hosts including humans, birds, and equines [8, 12–16, 30]. Furthermore, serological confirmations of DENV in humans were periodically reported in the southeast part of the country, close to the border with Pakistan [7, 19–21], but local transmission has not been confirmed yet [22]. Endemic to Pakistan since 1994, dengue fever caused a large-scale epidemic in 2006 [31]. In 2011, 20,864 cases were reported in Punjab province and 17,256 in Lahore with respectively 323 and 279 deaths [32].

Reports of CHIKV epidemics are recurrently made in countries neighboring Iran: Pakistan [23, 24], Qatar [33], Yemen [34–36], Iraq [37], Saudi Arabia [36, 37][38, 39], posing the threat of an emergence of CHIKV in Iran. CHIKV disseminates through displacements of viremic people and can extend locally through spreading of infected mosquitoes [40]. Additionally, other vertebrates such as monkeys [41], rodents, and birds can serve as viral reservoirs [42]. The main CHIKV vectors are *Aedes aegypti* and *Aedes albopictus* [43]. In Iran, *Ae*. *aegypti* has not been reported for more than 60 years [1] and *Ae*. *albopictus* is only present in the southeastern regions at the border with Pakistan [44, 45]. This latter is experimentally competent to 26 arboviruses [46] including CHIKV [43]. Against all expectations, this species has been involved in local transmission of dengue, chikungunya, and even Zika in Europe [47, 48]. Thus, even though *Ae*. *albopictus* is present at low densities and *Ae*. *aegypti* is still absent, the fear of an active circulation of these arboviruses is not negligible [49]. Besides, *Cx*. *annulirostris*, *Mansonia uniformis*, and *Anopheles* mosquitoes have also been occasionally incriminated [42–44]. Our screening method has detected CHIKV RNA in three species, *An*. *maculipennis* s.l., *Cs. longiareolata*, and *Cx*. *tritaeniorhynchus*. To the best of our knowledge, this is the first report of CHIKV infection in *Cx*. *tritaeniorhynchus* and *An*. *maculipennis* s.l. mosquitoes. *Cx*. *tritaeniorhynchus* and *An*. *maculipennis* s.l. have a wide zoogeographical distribution in Iran [49]. *An*. *maculipennis* s.l. is responsible to transmit human malaria parasites in the country [50]. This vector has also been reported to be infected with WNV [51]. *Cx*. *tritaeniorhynchus* is susceptible to Rift Valley fever virus (RVFV) and was found to be infected with RVFV in Saudi Arabia [52]. This vector has also been incriminated in Japanese encephalitis transmission cycle [53]. Both species could be infected following a blood meal on viremic travelers coming from Pakistan where CHIKV has been circulating since 2016 [54]. The circulating CHIKV belonged to the Asian genotype, as we have detected. Extending our study to southern regions at borders with DENV- and CHIKV-epidemic countries will help in tracking *Aedes*-transmitted arboviruses and implement appropriate measures to limit arboviral introductions in Iran. Our high-throughput screening method by targeting a large range of arboviruses has succeeded in detecting unexpected viruses. This method allows performing 9,216 real-time PCRs in a single run within four hours. The cost is quite low, around $10 per reaction (from RNA extraction to virus detection) [26]. Nevertheless, the instrument is still costly. However, this screening method can be adapted to different biological material (human and/or animal blood or organs) and include other arboviruses (e.g. tick-borne viruses). It can then be suggested with confidence in detecting arboviruses detrimental for human/animal health, especially in situations of viral emergences.

## Supporting information

S1 Table Details of identified mosquitoes collected from three provinces of Iran. (XLSX)

## References

[1] Azari-Hamidian S. Checklist of Iranian mosquitoes (Diptera: Culicidae). J Vector Ecol. 2007;32(2):235–42. 10.3376/1081-1710(2007)32[235:coimdc]2.0.co;2 18260513

[2] Azari-Hamidian S. Larval habitat characteristics of mosquitoes of the genus Culex (Diptera: Culicidae) in Guilan Province, Iran. Iran J Arthropod Borne Dis. 2007;1(1):9–20. PMC3385571 22808409

[3] Djadid ND, Jazayeri H, Gholizadeh S, Rad SP, Zakeri S. First record of a new member of Anopheles Hyrcanus Group from Iran: molecular identification, diagnosis, phylogeny, status of kdr resistance and Plasmodium infection. J Med Entomol. 2009;46(5):1084–93. 10.1603/033.046.0515 19769039

[4] Oshaghi M, Yaghobi-Ershadi M, Shemshad K, Pedram M, Amani H. The Anopheles superpictus complex: introduction of. Bull Soc Pathol Exot. 2008;101(5):429–34. 19192616

[5] Caragata EP, Tikhe CV, Dimopoulos G. Curious entanglements: interactions between mosquitoes, their microbiota, and arboviruses. Curr Opin Virol. 2019;37:26–36. 10.1016/j.coviro.2019.05.005 31176069 PMC6768729

[6] Smith DW. Arboviruses. Microbiol Aust. 2018;39(2):65–.

[7] Heydari M, Metanat M, Rouzbeh-Far M-A, Tabatabaei SM, Rakhshani M, Sepehri-Rad N, et al Dengue fever as an emerging infection in southeast Iran. Am J Trop Med Hyg. 2018;98(5):1469–71. 10.4269/ajtmh.17-0634 29557328 PMC5953366

[8] Ziyaeyan M, Behzadi MA, Leyva-Grado VH, Azizi K, Pouladfar G, Dorzaban H, et al Widespread circulation of West Nile virus, but not Zika virus in southern Iran. PLoS Negl Trop Dis. 2018;12(12):e0007022 10.1371/journal.pntd.0007022 30557321 PMC6312345

[9] Naficy K, Saidi S. Serological survey on viral antibodies in Iran. Trop Geogr Med. 1970;22(2):183–8. 4317129

[10] Saidi S, Tesh R, Javadian E, Nadim A. The prevalence of human infection with West Nile virus in Iran. Iran J Public Health. 1976;5(1):8–13.

[11] Sharifi Z, Mahmoudian SM, Talebian A. A study of West Nile virus infection in Iranian blood donors. Arch Iran Med. 2010;13(1):1–4. 20039761

[12] Ahmadnejad F, Otarod V, Fallah M, Lowenski S, Sedighi-Moghaddam R, Zavareh A, et al Spread of West Nile virus in Iran: a cross-sectional serosurvey in equines, 2008–2009. Epidemiol Infect. 2011;139(10):1587–93. 10.1017/S0950268811000173 21396143

[13] Fereidouni SR, Ziegler U, Linke S, Niedrig M, Modirrousta H, Hoffmann B, et al West Nile virus monitoring in migrating and resident water birds in Iran: are common coots the main reservoirs of the virus in wetlands? Vector Borne Zoonotic Dis. 2011;11(10):1377–81. 10.1089/vbz.2010.0244 21923253

[14] Chinikar S, Javadi A, Ataei B, Shakeri H, Moradi M, Mostafavi E, et al Detection of West Nile virus genome and specific antibodies in Iranian encephalitis patients. Epidemiol Infect. 2012;140(08):1525–9. 22008154 10.1017/S0950268811002056

[15] Chinikar S, Shah-Hosseini N, Mostafavi E, Moradi M, Khakifirouz S, Jalali T, et al Seroprevalence of West Nile virus in Iran. Vector Borne Zoonotic Dis. 2013;13(8):586–9. 10.1089/vbz.2012.1207 23697768

[16] Bagheri M, Terenius O, Oshaghi MA, Motazakker M, Asgari S, Dabiri F, et al West Nile virus in mosquitoes of Iranian wetlands. Vector Borne Zoonotic Dis. 2015;15(12):750–4. 10.1089/vbz.2015.1778 26565610

[17] Shahhosseini N, Chinikar S, Moosa‐Kazemi SH, Sedaghat MM, Kayedi MH, Lühken R, et al West Nile Virus lineage‐2 in culex specimens from Iran. Trop Med Int Health. 2017;22(10):1343–9. 10.1111/tmi.12935 28746985

[18] Bhatt S, Gething PW, Brady OJ, Messina JP, Farlow AW, Moyes CL, et al The global distribution and burden of dengue. Nature. 2013;496(7446):504 10.1038/nature12060 23563266 PMC3651993

[19] Aghaie A, Aaskov J, Chinikar S, Niedrig M, Banazadeh S, Mohammadpour HK. Frequency of dengue virus infection in blood donors in Sistan and Baluchestan province in Iran. Transfus Apher Sci. 2014;50(1):59–62. 10.1016/j.transci.2013.07.034 24332363

[20] Chinikar S, Ghiasi SM, Moradi A, Madihi SR. Laboratory Detection Facility of Dengue Fever (DF) in Iran: The First Imported Case. Int J Infect Dis. 2010;8(1):1–2.

[21] Mardani M, Abbasi F, Aghahasani M, Ghavam B. First Iranian imported case of dengue. Int J Prev Med. 2013;4(9):1075 24130951 PMC3793491

[22] Humphrey JM, Cleton NB, Reusken CB, Glesby MJ, Koopmans MP, Abu-Raddad LJ. Dengue in the Middle East and North Africa: a systematic review. PLoS Negl Trop Dis. 2016;10(12):e0005194 10.1371/journal.pntd.0005194 27926925 PMC5142774

[23] Rauf M, Manzoor S, Mehmood A, Bhatti S. Outbreak of chikungunya in Pakistan. Lancet Infect Dis. 2017;17(3):258. 10.1016/S1473-3099(17)30074-9 28244384

[24] Sahibzada HA, Khurshid Z, Khan RS, Zafar MS, Siddiqi KM. Outbreak of chikungunya virus in Karachi, Pakistan. J Ayub Med Coll Abbottabad. 2018;30(3):486–9. 30465393

[25] Horwood P, Buchy P. Chikungunya. Rev Sci Tech Off Int Epiz. 2015;34(2):479–89. 10.20506/rst.34.2.2373 26601450

[26] Moutailler S, Yousfi L, Mousson L, Devillers E, Vazeille M, Vega-Rua A, et al A New High-Throughput Tool to Screen Mosquito-Borne Viruses in Zika Virus Endemic/Epidemic Areas. Viruses. 2019;11(10). 10.3390/v11100904 31569736 PMC6832350

[27] Azari-Hamidian S, Harbach RE. Keys to the adult females and fourth-instar larvae of the mosquitoes of Iran (Diptera: Culicidae). Zootaxa. 2009;2078(1):1–33.

[28] Michelet L, Delannoy S, Devillers E, Umhang G, Aspan A, Juremalm M, et al High-throughput screening of tick-borne pathogens in Europe. Front Cell Infect Microbiol. 2014;29(4):103. 10.3389/fcimb.2014.00103 PMC4114295 25120960

[29] Eiras AE, Pires SF, Staunton KM, Paixao KS, Resende MC, Silva HA, et al A high-risk Zika and dengue transmission hub: virus detections in mosquitoes at a Brazilian university campus. Parasit Vectors. 2018;11(1):359 10.1186/s13071-018-2883-8 29929561 PMC6014031

[30] Meshkat Z, Chinikar S, Shakeri M, Manavifar L, Moradi M, Mirshahabi H, et al Prevalence of West Nile virus in Mashhad, Iran: A population–based study. Asian Pac J Trop Med. 2015;8(3):203–5. 10.1016/S1995-7645(14)60315-1 25902161

[31] Yousaf MZ, Siddique A, Ashfaq UA, Ali M. Scenario of dengue infection & its control in Pakistan: An up—date and way forward. Asian Pac J Trop Med. 2018;11(1):15–23.

[32] Qureshi A, Mahmood E, Tabinda AB, Vehra S, Yaqub A. Distribution and Seasonality of Horizontally Transmitted Dengue Viruses in Aedes Mosquitoes in a Metropolitan City Lahore, Pakistan. Pakistan J Zool. 2019;51(1):241–7.

[33] Humphrey JM, Al-Absi ES, Hamdan MM, Okasha SS, Al-Trmanini DM, El-Dous HG, et al Dengue and chikungunya seroprevalence among Qatari nationals and immigrants residing in Qatar. PloS one. 2019;14(1):e0211574 10.1371/journal.pone.0211574 30703150 PMC6355019

[34] Ciccozzi M, Presti AL, Cella E, Giovanetti M, Lai A, El-Sawaf G, et al Phylogeny of dengue and Chikungunya viruses in Al Hudayda governorate, Yemen. Infect Genet Evol. 2014;27:395–401. 10.1016/j.meegid.2014.08.010 25183027

[35] Fahmy NT, Klena JD, Mohamed AS, Zayed A, Villinski JT. Complete genome sequence of chikungunya virus isolated from an Aedes aegypti mosquito during an outbreak in Yemen, 2011. Genome Announc. 2015;3(4):e00789–15. 10.1128/genomeA.00789-15 26184944 PMC4505132

[36] Thabet A, Al-Eryani S, Aziz N, Obadi M, Saleh M, Al-Kohlani A. Epidemiological characterization of chikungunya outbreak in Lahj Governorate, Southern Yemen. J Community Med Health Educ. 2013;3(247):2161–0711.1000247.

[37] Barakat AM, Smura T, Kuivanen S, Huhtamo E, Kurkela S, Putkuri N, et al The presence and seroprevalence of arthropod-borne viruses in Nasiriyah governorate, southern Iraq: a cross-sectional study. Am J Trop Med Hyg. 2016;94(4):794–9. 10.4269/ajtmh.15-0622 26880770 PMC4824220

[38] Hussain R, Alomar I, Memish Z. Chikungunya virus: emergence of an arthritic arbovirus in Jeddah, Saudi Arabia. East Mediterr Health J. 2013;19(5):506–8. 24617133

[39] Syed A. Chikungunya Virus: An Infectious Disease. Int J Curr Res Biol Med. 2018;3(10):20–30.

[40] Morrison TE. Reemergence of chikungunya virus. J Virol. 2014;88(20):11644–7. 10.1128/JVI.01432-14 25078691 PMC4178719

[41] Apandi Y, Nazni W, Noor Azleen Z, Vythilingham I, Noorazian M. The first isolation of chikungunya virus from nonhuman primates in Malaysia. J Gen Mol Virol. 2009;1(3):35–9.

[42] Pialoux G, Gauzere BA, Jaureguiberry S, Strobel M. Chikungunya, an epidemic arbovirosis. Lancet Infectious Diseases. 2007;7(5):319–27. 10.1016/S1473-3099(07)70107-X . 17448935

[43] Reiter P, Fontenille D, Paupy C. Aedes albopictus as an epidemic vector of chikungunya virus: another emerging problem? Lancet Infect Dis. 2006;6(8):463–4. 10.1016/S1473-3099(06)70531-X 16870524

[44] Doosti S, Yaghoobi-Ershadi MR, Schaffner F, Moosa-Kazemi SH, Akbarzadeh K, Gooya MM, et al Mosquito surveillance and the first record of the invasive mosquito species Aedes (Stegomyia) albopictus (Skuse)(Diptera: Culicidae) in southern Iran. Iran J Public Health. 2016;45(8):1064–73. 27928533 PMC5139964

[45] Nejati J, Bueno-Marí R, Collantes F, Hanafi-Bojd AA, Vatandoost H, Charrahy Z, et al Potential risk areas of Aedes albopictus in South-Eastern Iran: a vector of Dengue Fever, Zika, and Chikungunya. Front Microbiol. 2017;8:1660 10.3389/fmicb.2017.01660 28928720 PMC5591785

[46] Paupy C, Delatte H, Bagny L, Corbel V, Fontenille D. Aedes albopictus, an arbovirus vector: from the darkness to the light. Microbes Infect. 2009;11(14–15):1177–85. 10.1016/j.micinf.2009.05.005 19450706

[47] Rezza G. Dengue and chikungunya: long-distance spread and outbreaks in naive areas. Pathog Glob Health. 2014;108(8):349–55. 10.1179/2047773214Y.0000000163 25491436 PMC4394667

[48] Giron S, Franke F, Decoppet A, Cadiou B, Travaglini T, Thirion L, et al Vector-borne transmission of Zika virus in Europe, southern France, August 2019. Euro Surveillance. 2019; 24(45):pii = 1900655. 10.2807/1560-7917.ES.2019.24.45.1900655. PMC6852313 31718742

[49] Azari-Hamidian S, Norouzi B, Harbach RE. A detailed review of the mosquitoes (Diptera: Culicidae) of Iran and their medical and veterinary importance. Acta trop. 2019;194:106–22. 10.1016/j.actatropica.2019.03.019 30898616

[50] Hanafi-Bojd AA, Azari-Hamidian S, Hassan V, Zabihollah C. Spatio-temporal distribution of malaria vectors (Diptera: Culicidae) across different climatic zones of Iran. Asian Pac J Trop Med. 2011;4(6):498–504. 10.1016/S1995-7645(11)60134-X 21771707

[51] Hubalek Z, Halouzka J. West Nile fever—a reemerging mosquito-borne viral disease in Europe. Emerg Infect Dis. 1999;5(5):643–50. 10.3201/eid0505.990505 10511520 PMC2627720

[52] Jupp P, Kemp A, Grobbelaar A, Leman P, Burt F, Alahmed A, et al The 2000 epidemic of Rift Valley fever in Saudi Arabia: mosquito vector studies. Med Vet Entomol. 2002;16(3):245–52. 10.1046/j.1365-2915.2002.00371.x 12243225

[53] Gould DJ, Edelman R, Grossman RA, Nisalak A, Sullivan MF. Study of Japanese encephalitis virus in ChiangMai valley, Thailand IV. Vector studies. Am J Epidemiol. 1974;100(1):49–56. 10.1093/oxfordjournals.aje.a112008 4842558

[54] Guhar D, Jamil N, Talpur SJ, Channa GA, Wajeeh M, Khan MZ, et al The 2016–2017 Chikungunya Outbreak in Karachi. PLoS Curr. 2018;10. 10.1371/currents.outbreaks.7257f6b05d8c18cf9e6eb222248be79f PMC6112271 30210935

